# A new software for fragment-based QSAR and its applications

**DOI:** 10.1186/1758-2946-6-S1-P11

**Published:** 2014-03-11

**Authors:** Sergey B Sosnin, Vladimir A Palyulin, Nikolay S Zefirov

**Affiliations:** 1 Department of Chemistry, Lomonosov Moscow State University, Moscow, 119991, Russia

## 

Fragment-based methods are quite popular in 2D QSAR/QSPR studies. In the advanced versions of these approaches for developing highly predictive models, one have to generate a huge set of descriptors that in turn requires well-designed algorithms and high-quality parallelism. To overcome these problems we developed the software for tagged generation of fragmental descriptors.

One of the most perspective programming paradigms is functional programming. Programs in pure functional languages are easy to parallelize, tend to be faultless, more clear, easy extensible [1]. Despite its tangible complexity for learning, functional programming is becoming rather popular, recently several chemoinformatics frameworks: OUCH and chemf were presented. We developed the program QLab for the fragmentation of molecular graphs in the imperative programming language (Java, using CDK), and ported it under the name FragmenT into the functional language (Haskell). Aware of the fact that graph operations is a core of chemoinformatics, FragmenT represents chemical structures by means of Functional Graph Library – a powerful tool for graph operations [2].

Using this software we processed several structure-activity/property databases containing hundreds of compounds and generated the sets of more than 500 000 fragmental descriptors in each case. Then for each database 100 most correlated with activity/property descriptors were selected using stagewise MLR. Based on selected descriptors the predictive models were developed using artificial neural networks (ANN) and Random Forest algorithms. Comparison with other fragmental descriptors generation software showed better predictive ability of our models.
